# Physical, Psychiatric, and Social Comorbidities of Individuals with Schizophrenia Living in the Community in Japan

**DOI:** 10.3390/ijerph20054336

**Published:** 2023-02-28

**Authors:** Masaaki Matsunaga, Yuanying Li, Yupeng He, Taro Kishi, Shinichi Tanihara, Nakao Iwata, Takahiro Tabuchi, Atsuhiko Ota

**Affiliations:** 1 Department of Public Health, Fujita Health University School of Medicine, Toyoake 470-1192, Japan; 2 Department of Public Health and Health Systems, Graduate School of Medicine, Nagoya University, Nagoya 466-8550, Japan; 3 Department of Psychiatry, School of Medicine, Fujita Health University, Toyoake 470-1192, Japan; 4 Department of Public Health, School of Medicine, Kurume University, Kurume 830-0011, Japan; 5 Cancer Control Center, Osaka International Cancer Institute, Osaka 541-8567, Japan

**Keywords:** community support, comorbidity, depression, epidemiology, schizophrenia

## Abstract

The physical, psychiatric, and social comorbidities interfere with the everyday activities of community-dwelling individuals with schizophrenia and increase the risk of their readmission. However, these comorbidities have not been investigated comprehensively in Japan. We conducted a self-reported internet survey in February 2022 to identify individuals aged 20–75 years with and without schizophrenia using a prevalence case-control study. The survey compared physical comorbidities such as being overweight, hypertension, and diabetes; psychiatric comorbidities such as depressive symptoms and sleep disturbances; social comorbidities such as employment status, household income, and social support between participants with and without schizophrenia. A total of 223 participants with schizophrenia and 1776 participants without schizophrenia were identified. Participants with schizophrenia were more likely to be overweight and had a higher prevalence of hypertension, diabetes, and dyslipidemia than participants without schizophrenia. Additionally, depressive symptoms, unemployment, and non-regular employment were more prevalent in participants with schizophrenia than those without schizophrenia. These results highlight the necessity of comprehensive support and interventions addressing physical, psychiatric, and social comorbidities in individuals with schizophrenia in the community. In conclusion, effective interventions for managing comorbidities in individuals with schizophrenia are necessary to enable them to continue to live in the community.

## 1. Introduction

Schizophrenia is a common illness with a reported lifetime prevalence of approximately 1% [1]. The World Health Organization’s Comprehensive Mental Health Action Plan 2013–2030 advocates for deinstitutionalization of individuals with schizophrenia, shifting the place of care from long-term inpatient psychiatric hospitals to non-specialized community-based health settings and providing comprehensive, integrated, and responsive mental health and social care. In Japan, approximately 150,000 individuals with schizophrenia are hospitalized, representing 60% of admissions for mental and behavioral disorders and 12% of all disease admissions [2]. Since the proposal of “The Vision for Reforming Mental Health Care and Welfare” in 2004, there has been a shift toward community-based care for individuals with schizophrenia [3]. Meanwhile, approximately 15–30% of individuals with schizophrenia are readmitted within 90 days of discharge worldwide [4]. Physical, psychiatric, and social comorbidities have been associated with readmission [5,6,7]. Investigating the physical, psychiatric, and social comorbidities of individuals with schizophrenia in the community can aid in identifying their needs and improving their care.

Evidence on physical, psychiatric, and social comorbidities associated with schizophrenia is accumulating [1]. For example, physical comorbidities include obesity [8], diabetes [9], hypertension [8], and hyperlipidemia [8]; psychiatric comorbidities include depression [10] and sleep disorder [11]; social comorbidities include low employment rate [12] and functional impairment such as community living and work [13]. In Japan, some comorbidities, such as overweight, hypertension, diabetes, depressive symptoms, quality of life, employment rate, and household income, have been reported [14,15]. However, there are no comprehensive reports on the physical, psychiatric, and social comorbidities of Japan’s community-dwelling individuals with schizophrenia [1].

Community-dwelling individuals with schizophrenia face unique challenges related to their physical, psychiatric, and social comorbidities. These comorbidities, such as obesity, depression, and low employment rate [16,17,18], can make it difficult for individuals with schizophrenia to function in everyday life. In addition, obesity is a risk factor for diabetes, and diabetes is one of the most significant mortality risk factors for individuals with schizophrenia [19]. Depression in individuals with schizophrenia can exacerbate the symptoms of schizophrenia, worsen the quality of life, and increase the risk of suicide [20]. Unemployment in individuals with schizophrenia can reduce the quality of life and place an extended burden on social support and disability services [21,22,23]. Addressing physical, psychiatric, and social comorbidities in primary care in the community is crucial in improving the health outcomes of individuals with schizophrenia.

To aid in the development of better treatment and support services for individuals with schizophrenia in the community, we conducted an internet survey to compare the prevalence of physical, psychiatric, and social comorbidities between individuals with and without schizophrenia living in the community in Japan.

## 2. Materials and Methods

### 2.1. Study Design and Participants

We conducted a prevalence case–control study using an internet research agency’s pooled panels (Rakuten Insight, which had approximately 2.3 million panelists in 2022). We collected data from those currently without schizophrenia and those currently with schizophrenia in February 2022.

For those currently without schizophrenia, we sampled 28,000 participants in the Japan Society and New Tobacco Internet Survey (JASTIS) [24] and the Japan COVID-19 and Society Internet Survey (JACSIS) [25,26,27] conducted by the Rakuten Insight Panel. Responses were obtained from 6656 respondents, who were asked the following questions before the survey. Those who answered no to all four questions were considered not to have schizophrenia: (1) Are you currently suffering from mental illness?; (2) Have you had mental illness in the past?; (3) Have you experienced auditory hallucinations?; (4) Have you ever used stimulants or other illegal drugs, been an alcoholic, or received psychiatric treatment? Finally, we obtained 1776 participants between the ages of 20 and 75 according to the sex and age structure of the Rakuten Insight Panel.

For those currently with schizophrenia, we sampled 5584 individuals aged 20 to 75 years who self-reported schizophrenia in the Rakuten Insight disease panel, a subset of the Rakuten Insight Panel. Responses were obtained from 3256 respondents, who were asked the following questions before the survey. Those who answered yes to all four questions were considered to currently have schizophrenia: (1) Are you currently suffering from schizophrenia only, schizophrenia and migraine, schizophrenia and a sleep disorder, or schizophrenia, migraine, and a sleep disorder?; (2) Have you experienced auditory hallucinations lasting more than one month?; (3) Have you never used stimulants or other illegal drugs and never been an alcoholic?; (4) Have you experienced the first auditory hallucination lasting more than one month at less than 60 years of age? A final response was received from 223 respondents.

### 2.2. Study Variables

A self-administered questionnaire assessed demographic and health-related backgrounds, physical comorbidities, psychiatric comorbidities, and social comorbidities.

Demographic and health-related backgrounds include age, body mass index (BMI) (underweight: <18.5 kg/m^2^, normal: 18.5–24.9 kg/m^2^, or overweight: ≥25.0 kg/m^2^), smoking status (current, past, or never), reason for quitting smoking (bad for health, illness, or other (e.g., financial reasons)), alcohol drinking (current drinker (≥23.0 g/day of ethanol), current drinker (<23.0 g/day ethanol), ex-drinker, or never drinker), reason for quit drinking (bad for health, illness, or other (e.g., financial reasons)), sports (<1 times per week or ≥1 times per week), tendency to overeat, eating speed (fast, normal, or slow), eating instant foods (<1 times per week, 1–4 times per week, or ≥5 times per week), bowel movement (<3 times per week, 3–7 times per week, or ≥2 times per day), stool (soft, normal, hard, or recurrent diarrhea and constipation), restriction in functional capacity, and bad self-rated health status (SRHS).

- Restriction in functional capacity.
  To evaluate functional capacity restrictions, we used the Scale of Independence in Daily Living for the Disabled Elderly published by the Ministry of Health, Labour and Welfare, Japan [28]. The term ”restrictions in functional capacity” refers to a multidimensional concept that involves sensory loss, impaired mobility, vascular disorders, gait impairments, problems with activities of daily living (ADLs), and changes in body systems [29]. Participants self-assessed restrictions by choosing one of the following options: (1) “I have no physical disabilities,” (2) “I go out alone, using transportation,” (3) “I can only go out alone in my neighborhood,” (4) “I go out with help and live mostly out of bed during the day,” (5) “I can go out with help, but I go out infrequently, and I spend most of the daytime sleeping on and off in bed,” (6) “I can ride in a wheelchair by myself and eat and toilet away from the bed,” (7) “I can ride in a wheelchair with assistance.,” (8) “I can roll over in bed,” and (9) “I cannot roll over in bed.” Participants who chose options other than option (1) were regarded as having restrictions in functional capacity. Participants who chose option (1), (2), or (3) were regarded as going out alone.
- Self-rated health status (SRHS).
  SRHS is a self-reported measure of health status that incorporates a person’s biological, mental, social, and functional aspects, including individual and cultural beliefs and health behaviors. It is a strong predictor of all-cause mortality in general populations [30]. Participants responded to the question “What do you think of your general health status during the previous month?” by choosing one of the following options: “great,” “pretty good,” “good,” “not so good,” and “bad.” Participants who answered “not so good” or “bad” were defined as bad SRHS.

Physical comorbidities include overweight, cancer, cardiovascular disease, heart failure, hypertension, diabetes, dyslipidemia, gout, sleep apnea syndrome, and fracture.

Psychiatric comorbidities include depressive symptoms (absent or present), sleep time (<5 h, 6–7 h, 8–9 h, or ≥10 h), hypnagogic disorder (<3 times per week or ≥3 times per week), deep sleep disorder (<3 times per week or ≥3 times per week), middle wakening or early wakening (<3 times per week or ≥3 times per week), perceived stress (absent or present), *ikigai* (absent or present), happiness (absent or present), and internet use time per week (h).

- Depressive symptoms (CES-D).
  We used a modified 11-item Center for Epidemiological Studies Depression (CES-D) Scale in this study [31,32]. The existence of depressive symptoms was defined as a score of 8 or higher.
- Hypnagogic disorder.
  Participants were asked, “In the past month, have you had trouble falling asleep within 30 min of getting to bed?” and answered the question from six options: “almost never,” “less than once a week,” “1–2 times per week,” “3–4 times per week,” “5–6 times a week,” or “almost every day.” Participants who answered “3–4 times per week,” “5–6 times a week,” or “almost every day” were defined as having hypnagogic disorder.
- Deep sleep disorder.
  Participants were asked, “In the past month, have you felt terribly tired when you woke up in the morning?” and answered the question from six options: “almost never,” “less than once a week,” “1–2 times per week,” “3–4 times per week,” “5–6 times a week,” or “almost every day.” Participants who answered “3–4 times per week,” “5–6 times a week,” or “almost every day” were defined as having deep sleep disorder.
- Perceived stress (PSS-4).
  We assessed perceived stress with a 4-item Perceived Stress Scale (PSS-4) [33]. Scores are on a 16-point scale, with higher total scores indicating more severe perceived stress. Perceived stress was defined as being present when the score was higher than 7, the median of the PSS-4 scores for participants without schizophrenia.
- *Ikigai.*
  The Japanese term “*Ikigai*” is a positive reason for living [34]. Participants were asked, “Do you have any positive reasons to live?” and answered the question from four options: “very much so,” “yes,” “no,” or “not at all.” Participants who answered “no” or “not at all” were defined as the absence of *ikigai*.
- Happiness.
  Participants were asked, “How happy do you feel about your life?” and answered the question from four options: “very happy,” “happy,” “neither happy nor unhappy,” and “unhappy.” Participants who answered “neither happy nor unhappy” or “unhappy” were defined as the absence of happiness.
- Internet use time.
  Internet use time per week was calculated from the hours of use per day and the frequency of use per week. We defined longtime internet use as more than 14 h, a median of participants without schizophrenia.

Social comorbidities include taking regular medical checkups, educational background (junior/senior high school, university, junior college, or vocational school), occupation (unemployed, homemaker, white-collar workers, or blue-collar workers), type of employment (regular, non-regular, or self-employed/business people), household income (million Japanese yen) (<3, 3–6, 6–9, or ≥9), marital status (unmarried, married, divorced, widowed, or others), family structure (living alone, living with parents, living with spouse, living with children, and living with other people), social support, and social capital (cognitive and structural dimensions).

- Non-regular employment.
  A regular employee in Japan is a term used to refer to an employee who does not have a set term of employment, works during scheduled hours, and is employed directly by his or her employer. A non-regular employee in Japan is an employee who does not meet one of the conditions for regular employment. In other words, non-regular employment falls within one or more fixed-term, part-time, or indirect employment.
- Social support.
  Social support was assessed using the ENRICHD Social Support Instrument (ESSI), which is a well-validated and widely used self-report questionnaire designed to assess the availability of social support [35,36]. The ESSI consists of 7 items that assess the perceived availability of social support in different domains, including emotional, instrumental, informational, and appraisal support. The 7 items of ESSI are as follows: (1) “Is there someone available you can count on to listen to you when you need to talk?,” (2) “Is there someone available to you to give you good advice about a problem?,” (3) “Is there someone available to you who shows you love and affection?,” (4) “Is there someone available to help with daily chores?,” (5) “Can you count on anyone to provide you with emotional support (for instance, talking over problems or helping you make a difficult decision)?,” (6) “Do you have as much contact as you would like with someone you feel close to and you can trust and confide in?,” and (7) “Are you living with your spouse or partner?” For the first six items, participants selected one of the following options: “none (score = 1),” “a little (score = 2),” “some (score = 3),” “most (score = 4),” and “all of the time (score = 5).” For item 7, participants who lived with their spouse or partner received a score of 4 and those who did not received a score of 2. Total scores ranged from 8 to 34, with higher scores indicating higher availability of social support.
  In the present study, we defined social support as low when the total score on the ESSI was less than 17, which corresponds to the first quartile of the ESSI scores for participants without schizophrenia.
- Social capital.
  Social capital was assessed using the Integrated Questionnaire for the Measurement of Social Capital (SC-IQ) [37], which is a comprehensive and multidimensional self-report questionnaire designed to measure different aspects of social capital, typically described as assets such as social networks, social participation, trust, and reciprocity. In the present study, we focused on cognitive and structural social capital using specific items from the SC-IQ [38]. For cognitive social capital, we used the following three items: (1) “Can most people be trusted?”; (2) “Does one have to be alert or is someone likely to take advantage of you?”; (3) “Are most people willing to help if you need it?” Responses were selected from four categories: “strongly disagree,” “disagree,” “agree,” and “strongly agree.” For the three questions, cognitive social capital was defined as high when there were two or more responses of “agree” or “strongly agree” to question (1), “disagree” or “strongly disagree” to question (2), and “agree” or “strongly agree” to question (3). For structural social capital, we used the following item from the SC-IQ: “How often do you participate in community organizations, self-help groups, charities, volunteer groups, or religious gatherings?” The response was selected from four categories: “not at all/very seldom,” “sometimes,” “less than once a week,” and “more than once a week.” Structural social capital was defined as high when the response was “more than once a week.”

### 2.3. Statistical Analysis

*T*-tests were used to compare the averages of continuous variables, and Fisher’s exact tests were used to compare the proportions of categorical variables between participants with and without schizophrenia.

A logistic regression analysis was used to calculate sex- and age-adjusted odds ratios (AORs) and 95% confidence intervals (CIs) of participants with schizophrenia compared to participants without schizophrenia for physical comorbidities, psychiatric comorbidities, and social comorbidities. All the analyses were conducted using R4.2.1 software (R Foundation: Vienna, Austria). The level of significance was set at *p* < 0.05 (two-sided).

## 3. Results

The study presented in Table 1 provides a comparison of demographic and health-related characteristics of participants with and without schizophrenia. Males with schizophrenia were more prevalent (52%) than females with schizophrenia. Overall, males were older than females, but there was no significant difference in age between participants with and without schizophrenia for males. On the other hand, women with schizophrenia were older than women without schizophrenia.

A higher percentage of participants with schizophrenia were overweight (BMI ≥ 25) in both sexes (53% for participants with schizophrenia vs. 28% for participants without schizophrenia in men and 39% vs. 9.3% in women, respectively). Smoking was more prevalent among women with schizophrenia. Fewer participants with schizophrenia were drinkers, and more participants with schizophrenia were abstinent drinkers compared to participants without schizophrenia. Eating habits were also compared between the two groups. Overeating was more prevalent among female participants with schizophrenia. Speed-eating was more prevalent among participants with schizophrenia. Additionally, participants with schizophrenia tended to consume instant foods more frequently. Bowel movements were also compared, and it was found that participants with schizophrenia had more frequent bowel movements and soft stools than participants without schizophrenia.

Participants with schizophrenia reported a higher level of functional restriction and bad self-rated health status than participants without schizophrenia. The percentage of those who felt a restriction in functional capacity was small in participants without schizophrenia (7.4% in men and 4.6% in women). In comparison, that percentage was about 40% in participants with schizophrenia (39% in men and 41% in women). A total of 94% of participants with schizophrenia went out daily. Similarly, the proportion of bad self-rated health status was higher among participants with schizophrenia than participants without schizophrenia.

As for physical diseases in participants with schizophrenia, in men, hypertension (18%), diabetes (14%), and dyslipidemia (13%) were common. In women, dyslipidemia (11%), hypertension (7.4%), and diabetes/cancer (6.5%) were common (Table 2).

**Figure 1:**
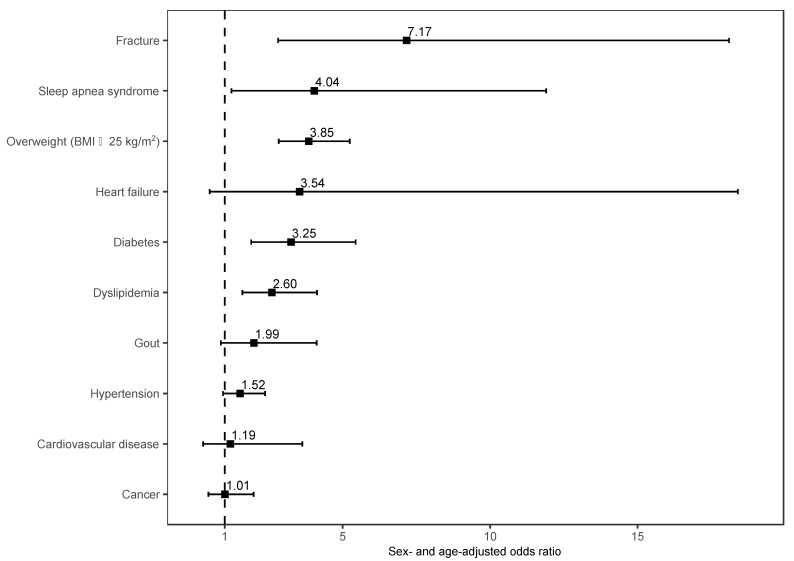
Sex- and age-adjusted odds ratios of participants with schizophrenia compared to participants without schizophrenia in terms of physical comorbidities. Abbreviations: BMI, body mass index. The reference group for overweight is normal BMI (18.5–24.9 kg/m^2^).

Figure 1 shows the results of a sex- and age-adjusted logistic regression model investigating the association between schizophrenia and physical comorbidities. Compared to community dwellers without schizophrenia, community dwellers with schizophrenia more frequently reported a history of fracture (AOR: 7.17, 95% CI = 2.81 to 18.1), sleep apnea syndrome (AOR: 4.04, 95% CI = 1.23 to 11.9), overweight (AOR: 3.85, 95% CI = 2.83 to 5.24), diabetes (AOR: 3.25, 95% CI = 1.90 to 5.44), and dyslipidemia (AOR: 2.60, 95% CI = 1.60 to 4.13).

Table 3 shows the psychiatric comorbidities of participants with and without schizophrenia. Participants with schizophrenia had more severe depressive symptoms (CES-D ≥ 8) and were more predominant among participants with schizophrenia compared to participants without schizophrenia (63% for participants with schizophrenia vs. 23% for participants without schizophrenia in men; 77% vs. 29% in women).

In terms of sleep patterns, participants with schizophrenia reported sleeping longer. They were more likely to report sleep disturbances, including hypnagogic disorder, deep sleep disorder, and middle or early awakenings, compared to participants without schizophrenia.

Perceived stress (PSS-4 scores) was higher in participants with schizophrenia than in participants without schizophrenia. More participants with schizophrenia had an absence of *ikigai* (a positive reason for living) and absence of happiness than participants without schizophrenia. Internet use was significantly longer in participants with schizophrenia compared to participants without schizophrenia in men, while no significant differences were found in women.

**Figure 2:**
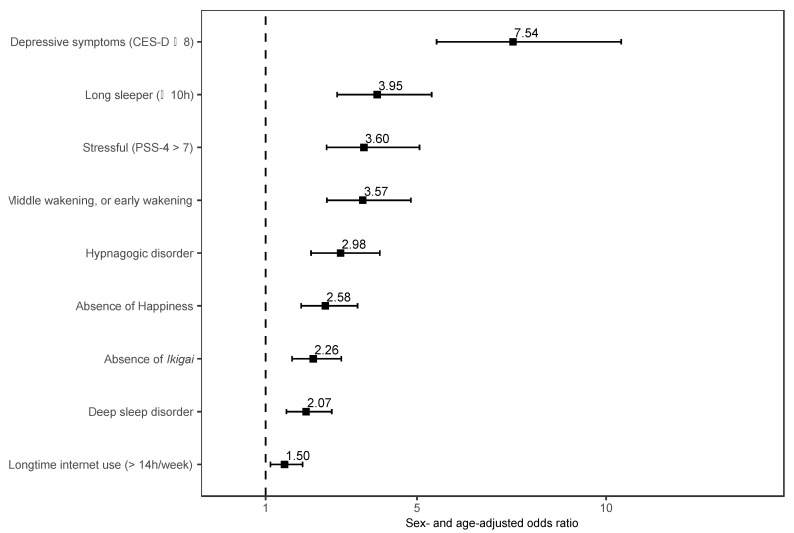
Sex- and age-adjusted odds ratios of participants with schizophrenia compared to participants without schizophrenia in terms of psychiatric comorbidities. Abbreviations: CES-D, a modified 11-item Center for Epidemiological Studies Depression Scale; PSS-4, a 4-item Perceived Stress Scale.

Figure 2 shows the results of a sex- and age-adjusted logistic regression model investigating the association between psychiatric comorbidities and schizophrenia. Depressive symptoms (CES-D ≥ 8) were associated with more strongly than other psychiatric comorbidities (AOR: 7.54, 95% CI = 5.52 to 10.4). Compared to community dwellers without schizophrenia, community dwellers with schizophrenia more frequently reported long-hour sleep (≥10 h) (AOR: 3.95, 95% CI = 2.89 to 5.39), stressful (PSS-4 > 7) (AOR: 3.60, 95% CI = 2.61 to 5.07), middle wakening or early wakening (AOR: 3.57, 95% CI = 2.62 to 4.84), hypnagogic disorder (AOR: 2.98, 95% CI = 2.20 to 4.02), absence of happiness (AOR: 2.58, 95% CI = 1.94 to 3.43), absence of *ikigai* (AOR: 2.26, 95% CI = 1.70 to 3.00), deep sleep disorder (AOR: 2.07, 95% CI = 1.55 to 2.75), and longtime internet use (>14 h per week) (AOR: 1.50, 95% CI = 1.13 to 1.98).

Table 4 shows the social comorbidities of participants with and without schizophrenia. Regarding health literacy and behaviors, participants with schizophrenia did not have regular health examinations compared to participants without schizophrenia. In terms of socioeconomic status, compared to participants without schizophrenia, participants with schizophrenia were less educated (junior/senior high school), unemployed (50% vs. 15% in men; 33% vs. 6.8% in women), non-regular employment (56% vs. 15% in men; 79% vs. 42% in women), low household income (<3 million Japanese yen) (53% vs. 18% in men; 44% vs. 22% in women), unmarried (especially among men), and living with parents. Regarding social support and social capital, the differences in ESSI scores with and without schizophrenia were small. Participants with schizophrenia had a lower cognitive social capital compared to participants without schizophrenia.

**Figure 3:**
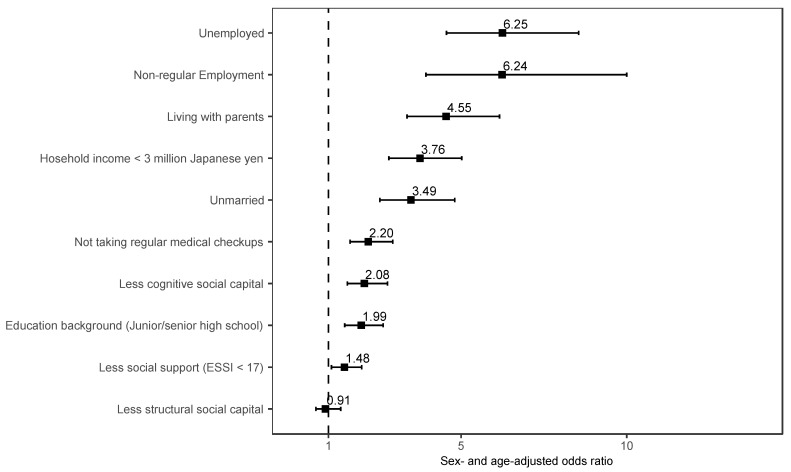
Sex- and age-adjusted odds ratios of participants with schizophrenia compared to participants without schizophrenia in terms of social comorbidities. Abbreviations: ESSI, the ENRICHD Social Support Instrument.

Figure 3 shows the results of a sex- and age-adjusted logistic regression model investigating the association between schizophrenia and social comorbidities. Unemployment (AOR: 6.25, 95% CI = 4.56 to 8.55) and non-regular employment (AOR: 6.24, 95% CI = 3.94 to 10.0) were significantly more strongly associated with schizophrenia than other social comorbidities. For other social comorbidities, compared to community dwellers without schizophrenia, community dwellers with schizophrenia more frequently reported living with parents (AOR: 4.55, 95% CI = 3.37 to 6.16), low household income (<3 million Japanese yen) (AOR: 3.76, 95% CI = 2.82 to 5.02), unmarried (AOR: 3.53, 95% CI = 2.58 to 4.84), not taking regular medical checkups (AOR: 2.20, 95% CI = 1.65 to 2.94), less cognitive social capital (AOR: 2.08, 95% CI = 1.57 to 2.78), low education background (Junior/senior high school) (AOR: 1.99, 95% CI = 1.49 to 2.65), and less social support (ESSI < 17) (AOR: 1.48, 95% CI = 1.09 to 2.00).

## 4. Discussion

This study revealed the physical, psychiatric, and social comorbidities of individuals with schizophrenia living in the community in Japan, which were seldom reported comprehensively.

The mean age of participants with schizophrenia in this study was 48 years for males and 44 years for females, which is consistent with the mean age of 42.7 years reported in The Japan National Health and Wellness Survey [15]. However, it should be noted that this estimated age of participants with schizophrenia may be younger than the mean age of individuals with schizophrenia in Japan. This is due to the fact that participants in this study were recruited via the internet, which could lead to a higher representation of younger adults with high internet usage [39]. Additionally, this recruitment method may not include hospitalized patients, many of whom are elderly.

Participants with schizophrenia had a higher percentage (53% of men and 39% of women) with a BMI of 25 or higher than participants without schizophrenia. The prevalence of BMI ≥ 25.0 kg/m^2^ among Japanese outpatients with schizophrenia has been reported to be 48.9% [14]. Individuals with schizophrenia have a shorter life expectancy due to death from cardiovascular causes [40,41]. Previous studies have also shown that outpatients have a higher rate of obesity than inpatients [14], highlighting the need for measures to control obesity in individuals with schizophrenia living in the community.

Approximately 10% of participants with schizophrenia in this study defecated less than three times per week, a prevalence lower than that reported in outpatients in Finland (31.3%) [42] and inpatients in Japan (36.6%) [43]. One potential explanation for this discrepancy may be that participants with schizophrenia had more than two bowel movements per day and a higher percentage of soft stools than those without schizophrenia, some of whom may be constipated. Antipsychotic medications are known to cause constipation, and it has been reported that over 50% of individuals with schizophrenia taking antipsychotic drugs experience constipation [44]. Many participants in this study likely took laxatives, such as osmotic laxatives such as magnesium hydroxide, which can soften stools. However, the anticholinergic effect of antipsychotics may reduce peristalsis in the bowel, leading to constipation with limited stool volume and a feeling of incomplete defecation.

Participants with schizophrenia have been found to engage in overeating and faster eating patterns compared to those without schizophrenia. Overeating may be due to increased appetite caused by the side-effects of antipsychotic medications [45]. In systematic reviews, the prevalence of binge eating among individuals with schizophrenia taking antipsychotic medications ranges from 4.4% to 45%, with the majority of participants being of Western origin [46]. As far as we know, no studies have compared the prevalence of eating fast in individuals with schizophrenia with that of the general population. Eating fast has been reported to be associated with obesity [47]. In clinical practice, it is crucial to instruct individuals with schizophrenia on appropriate food intake in terms of both quantity and speed to prevent obesity.

A comparison of smoking rates between male participants with schizophrenia and those without schizophrenia revealed no significant differences. However, female participants with schizophrenia were found to have smoking rates that were approximately 2.5 times higher than those without schizophrenia. Similarly, a meta-analysis of smoking rates among Japanese individuals with schizophrenia showed that compared to the general population, male individuals with schizophrenia had an odds ratio 1.53 times higher for smoking rates (52.9% for individuals with schizophrenia and 40.1% for the general population) and female individuals with schizophrenia had an odds ratio 2.40 times higher for smoking rates among females (24.4% for individuals with schizophrenia and 11.8% for the general population) [48]. This disparity in smoking rates may be attributed to the fact that the smoking rate in the Japanese population has been decreasing over time among men, but the decline is less pronounced among women [49].

The primary physical diseases among participants with schizophrenia were hypertension, diabetes mellitus, and dyslipidemia, all of which are associated with obesity. The prevalence of these diseases was relatively low compared to a previous study conducted in Japan [14], which may be attributed to the younger age of the participants in this study. However, the prevalence of diabetes mellitus and dyslipidemia was significantly higher than that of participants without schizophrenia. These diseases, as well as obesity, require caution when examining individuals with schizophrenia.

Although the absolute prevalence of fracture and sleep apnea was low, both conditions had high sex- and age-adjusted odds ratios for the prevalence of schizophrenia. Fracture is associated with antipsychotic medications, analgesics, and physical diseases such as hypertension in individuals with schizophrenia [50,51]. It has been reported that bone mineral density is decreased in Japanese outpatients with schizophrenia [52], highlighting the need for osteoporosis prevention. A meta-analysis reported a comorbidity of obstructive sleep apnea as high as 15.4% in schizophrenia [53]. In a Japanese survey, 19% of hospitalized individuals with schizophrenia had sleep apnea [54]. Because the medical history was self-reported, there may be undiagnosed obstructive sleep apnea. Potentially, the prevalence of obstructive sleep apnea could be even higher. Early detection and intervention for obstructive sleep apnea are needed to protect against sleep disturbance and cardiovascular disease [55].

Cardiovascular disease and heart failure, associated with schizophrenia in previous studies of Westerners [56,57], were not associated with schizophrenia in the present study. This may be partly because these diseases typically have an elderly onset and the sample size of participants with schizophrenia in the study was small. The present study also found no association between gout and schizophrenia, which is consistent with a systematic review and meta-analysis that found no difference in uric acid levels between individuals with and without schizophrenia [58]. Cancer was not associated with schizophrenia in this study, although the prevalence of cancer was higher in female participants with schizophrenia than in female participants without schizophrenia. This result is consistent with previous findings that schizophrenia is associated with a higher risk of breast cancer [59], although the incidence of cancer in individuals with schizophrenia has been reported to vary in comparison to the general population [60].

Depressive symptoms were present in more than two-thirds of participants with schizophrenia, making it the greatest risk factor for psychiatric comorbidities in schizophrenia (AOR: 7.54). This prevalence is higher than that reported in Japan’s National Health and Wellness Survey, where 47.8% of individuals with schizophrenia had depressive symptoms (85/178) [15]. The lifetime prevalence of depression in individuals with schizophrenia ranges from 16 to 69%, depending on factors such as the definition of depression, patient setting, and period of observation [10]. This is higher than that in the general population, which is consistent with our findings [17]. Depression in schizophrenia has been reported to be associated with factors that interfere with living in the community, such as schizophrenia relapse, early rehospitalization, impairment of social and occupational functioning, and family and community burden [10,61]. Additionally, depression in individuals with schizophrenia is strongly associated with an increased risk of suicide [62]. Therefore, addressing depressive symptoms is a crucial intervention for individuals with schizophrenia.

Participants with schizophrenia reported longer sleep duration and more sleep disturbances than those without schizophrenia. A systematic review reported that those with remitted schizophrenia showed a longer sleep duration, time in bed, and sleep latency than the healthy control did [63], which is consistent with our results. Individuals with schizophrenia complain of sleep disturbances not only in the acute phase but also in the remission phase [64]. In a Japanese study, the prevalence of individuals with schizophrenia with any sleep disturbances was 49.4% (88/178) [15]. In Chinese outpatients, the prevalence of at least one type of insomnia was 28.9% (180/623), while those with difficulty initiating sleep, difficulty maintaining sleep, and early morning wakening were 20.5%, 19.6%, and 17.7%, respectively [65]. The participants with schizophrenia in this study, most presumed outpatients, showed sleep disturbances at a higher rate compared to the previous research in China. Further studies are needed in different populations. Sleep disturbances tend to precede the onset of schizophrenia, and management of sleep disturbances can prevent acute exacerbation of psychiatric symptoms [63].

Perceived stress was stronger in participants with schizophrenia than in those without schizophrenia, which is consistent with previous evidence in Western populations [66,67]. A common finding is an association between stress and pathophysiology in all stages of schizophrenia [66]. Appropriate coping with stress is associated with improved quality of life in individuals with schizophrenia [68].

Participants with schizophrenia had less *ikigai* and happiness than participants without schizophrenia, which is consistent with less well-being, happiness, and life satisfaction in individuals with schizophrenia among Westerners [67,69]. However, the difference between young adult individuals with schizophrenia and the general population in subjective well-being scores is small [69]. In an interview survey conducted with mentally disabled persons living in a community in Japan, some of them realized *ikigai* through dialogue with interviewees [70]. *Ikigai* or happiness may vary depending on the patient background or may be difficult to realize in individuals with schizophrenia.

Individuals with schizophrenia were reported to spend more time using the internet compared to those without schizophrenia, especially in men. In South Korea, 22% of individuals with schizophrenia were reported to suffer from problematic internet use, which was associated with higher levels of perceived stress and lower coping skills [71].

Participants with schizophrenia did not receive regular medical checkups as compared to participants without schizophrenia. This result is consistent with reports that Korean people with psychosis demonstrated lower knowledge of physical illnesses and did not receive regular medical checkups [72]. Therefore, individuals with schizophrenia must be educated and encouraged to undergo medical checkups.

A higher percentage of participants with schizophrenia were unemployed or had non-regular employment, had lower household incomes, were less likely to be married, and lived with their parents than participants without schizophrenia. Despite evidence indicating that individuals diagnosed with schizophrenia are more likely to be unemployed [22] or have lower income [73] than the general population, there is a paucity of research investigating whether they are more likely to be unmarried or residing with their parents. However, our finding is consistent with a previous Japanese study, which also found a high prevalence of unmarried, unemployed, and low household income among individuals with schizophrenia [15]. Additionally, the number of claims for mental and behavioral disorders per population was lower in the Japanese Medical Data Center (JMDC) database, consisting of corporate health insurance claims, compared to the National Database (NDB), consisting of all claims data constructed by the Japanese government [74]. This aligns with the high percentage of non-regular employment among participants with schizophrenia in the present study.

Sociodemographic features specific to individuals with schizophrenia are interrelated. A survey on family support for individuals with mental disorders in Japan found that 85% of respondents were parents [75], indicating that many individuals with schizophrenia are unmarried and live under parental support. Furthermore, the patients reported that they were unemployed or had non-regular employment, and their household income was low. In addition to the patient’s work arrangement, family members are expected to work fewer hours to support the patient’s daily needs, resulting in lower household income. With the parents’ aging, further measures are needed to ensure that individuals with schizophrenia can continue to live in the community because the parents are concerned about livelihood support (74.8%) and financial aspects (60.1%) after the parents’ death [75]. From a medical and social perspective, there is a need for educational programs that can help individuals with schizophrenia support themself while also managing their mental health or programs that can help parents better understand the condition and how to support their children with schizophrenia.

Social support tended to be lower among participants with schizophrenia than those without schizophrenia. Cognitive social capital was significantly lower in participants with schizophrenia than in participants without schizophrenia, while structural social capital did not differ between participants with and without schizophrenia. It has been reported in Westerners that schizophrenia was associated with low social support [76] and low cognitive social capital at the ecological level [77], which is consistent with the results of this study. Community development from the perspective of social support and social capital is required to improve community residents’ mental health.

The present study has several strengths and limitations. First, online surveys may be susceptible to sampling bias and response bias, compared to population-based surveys. However, we did not use a stratified sampling technique to obtain as many responses as possible from respondents with schizophrenia. Second, the diagnosis of schizophrenia in this study was based on self-reports, which may limit the accuracy of the diagnosis. To address this limitation, we asked preliminary questions based on the DSM-5 diagnostic criteria [78] to exclude psychiatric disorders other than schizophrenia, such as depression, delusional disorder, and alcoholism, and to increase the specificity of the self-reported schizophrenia status. However, the sensitivity of the self-report survey may be low due to the lack of insight that often accompanies schizophrenia [79], potentially leading to an underestimation of the prevalence of the condition. In future studies, we plan to consider alternative approaches for assessing schizophrenia, such as clinical interviews or medical records, to improve the validity of our results. Third, the potential for underestimation of the prevalence of physical conditions among individuals with schizophrenia is due to the self-reported nature of the data, which can be influenced by cognitive deficits and low health literacy. Fourth, some of the participants with schizophrenia might not be living in the community, because we did not collect data about whether they lived there. This misclassification may cause the prevalence of comorbidities in schizophrenia to be biased, while the estimate of 94% with schizophrenia being out daily would support that most of the participants with schizophrenia live in the community. In addition, due to low health literacy, their comorbidities may be underreported. A more focused sample of community-dwelling individuals with confirmed psychiatric and medical diagnoses would be needed in future research. Fifth, the study design did not adequately include self-reflective components critical to understanding the daily experiences and perceptions of individuals with schizophrenia. Future research should consider self-reflective components because self-reflection could influence the perception of comorbidities. Sixth, the study has not collected sufficient data on other confounding variables, such as medication and menopause, that may have influenced the outcomes. Hence, in future research, it is imperative to collect data on potential confounding variables associated with the identified risk factors for schizophrenia. This would allow for a more comprehensive understanding of the underlying factors and their association with schizophrenia. Finally, this study was cross-sectional, and causal relationships must be carefully evaluated.

## 5. Conclusions

This study provides an overall description of comorbidities in individuals with schizophrenia living in the community in Japan using an internet survey. Physical comorbidities included overweight, hypertension, dyslipidemia, and diabetes. As for psychiatric comorbidities, depressive symptoms and sleep disorders were common. Social comorbidities included low education, unemployment/non-regular employment, low income, and living with parents. These findings suggest that a comprehensive approach is necessary to manage the physical, psychiatric, and social comorbidities in individuals with schizophrenia to continue to live in the community. The interventions should include lifestyle modifications, psychological therapies, vocational rehabilitation programs, job coaching, and supported employment programs. Such interventions can help individuals with schizophrenia to manage their comorbidities, improve their overall health outcomes and quality of life, and reduce the risk of readmission to hospitals. Healthcare providers, policymakers, and other stakeholders should work together to develop and implement interventions that address these comorbidities and improve the health outcomes and overall quality of life of individuals with schizophrenia living in the community.

## Figures and Tables

**Table 1:** Demographic and health-related backgrounds of participants with schizophrenia and participants without schizophrenia according to sex.

Characteristic	Men			Women		
	Schizophrenic Participants, N = 115	Non-Schizophrenic Participants, N = 801	*p*-Value	Schizophrenic Participants, N = 108	Non-Schizophrenic Participants, N = 975	*p*-Value
Age	48 (9)	48 (13)	0.651	44 (10)	42 (13)	0.014
Body mass index (kg/m^2)^			<0.001			<0.001
≥25	61 (53%)	222 (28%)		42 (39%)	91 (9.3%)	
18.5–24.9	52 (45%)	539 (67%)		56 (52%)	691 (71%)	
<18.5	2 (1.7%)	40 (5.0%)		10 (9.3%)	193 (20%)	
Smoking			0.699			<0.001
Current	32 (28%)	231 (29%)		21 (19%)	73 (7.5%)	
Never	52 (45%)	383 (48%)		76 (70%)	802 (82%)	
Past	31 (27%)	187 (23%)		11 (10%)	100 (10%)	
Reason for quitting smoking of past smoker			0.041			0.231
Bad for health	12 (39%)	113 (60%)		5 (45%)	55 (55%)	
Illness	3 (9.7%)	19 (10%)		2 (18%)	5 (5.0%)	
Other (e.g., financial reasons)	16 (52%)	55 (29%)		4 (36%)	40 (40%)	
Alcohol			<0.001			0.052
Never drinker	53 (46%)	338 (42%)		68 (63%)	599 (61%)	
Ex-drinker	32 (28%)	40 (5.0%)		22 (20%)	123 (13%)	
Current drinker (<23 ethanol g/day)	17 (15%)	190 (24%)		11 (10%)	171 (18%)	
Current drinker (≥23 ethanol g/day)	13 (11%)	233 (29%)		7 (6.5%)	82 (8.4%)	
Reason for quitting drinking of Ex-drinker			0.502			0.008
Bad for health	12 (38%)	20 (50%)		6 (27%)	46 (37%)	
Illness	8 (25%)	6 (15%)		6 (27%)	6 (4.9%)	
Other (e.g., financial reasons)	12 (38%)	14 (35%)		10 (45%)	71 (58%)	
Sports			0.072			0.918
<1 times per week	70 (61%)	413 (52%)		62 (57%)	568 (58%)	
≥1 times per week	45 (39%)	388 (48%)		46 (43%)	407 (42%)	
Tendency to overeat	66 (57%)	406 (51%)	0.195	74 (69%)	569 (58%)	0.049
Eating speed			<0.001			0.005
Fast	82 (71%)	404 (50%)		62 (57%)	399 (41%)	
Normal	19 (17%)	318 (40%)		32 (30%)	419 (43%)	
Slow	14 (12%)	79 (9.9%)		14 (13%)	157 (16%)	
Eating instant foods			0.191			0.206
<1 times per week	57 (50%)	459 (57%)		72 (67%)	672 (69%)	
1–4 times per week	49 (43%)	302 (38%)		30 (28%)	279 (29%)	
≥5 times per week	9 (7.8%)	40 (5.0%)		6 (5.6%)	24 (2.5%)	
Bowel motion			0.190			0.037
<3 times per week	10 (8.7%)	47 (5.9%)		11 (10%)	118 (12%)	
3–7 times per week	79 (69%)	608 (76%)		83 (77%)	798 (82%)	
≥2 times per day	26 (23%)	146 (18%)		14 (13%)	59 (6.1%)	
Stool			<0.001			<0.001
Soft	39 (34%)	145 (18%)		25 (23%)	86 (8.8%)	
Normal	59 (51%)	575 (72%)		63 (58%)	726 (74%)	
Hard	12 (10%)	64 (8.0%)		13 (12%)	131 (13%)	
Recurrent diarrhea and constipation	5 (4.3%)	17 (2.1%)		7 (6.5%)	32 (3.3%)	
Restrictions in functional capacity	45 (39%)	59 (7.4%)	<0.001	44 (41%)	45 (4.6%)	<0.001
Self-rated health status			<0.001			<0.001
Bad	51 (44%)	136 (17%)		57 (53%)	142 (15%)	
Not bad	64 (56%)	665 (83%)		51 (47%)	833 (85%)	

Continuous variables were expressed as mean (SD), and categorical variables as number (percentage). *p*-values were calculated with the Welch Two-Sample *t*-test for continuous variables and with Fisher’s exact test for categorical variables.

**Table 2:** Physical comorbidities of participants with schizophrenia and participants without schizophrenia according to sex.

Characteristic	Men			Women		
	Schizophrenic Participants, N = 115	Non-Schizophrenic Participants, N = 801	*p*-Value	Schizophrenic Participants, N = 108	Non-Schizophrenic Participants, N = 975	*p*-Value
Cancer	2 (1.7%)	30 (3.7%)	0.415	7 (6.5%)	36 (3.7%)	0.187
Cardiovascular disease	3 (2.6%)	13 (1.6%)	0.440	0 (0%)	4 (0.4%)	>0.999
Heart failure	1 (0.9%)	3 (0.4%)	0.416	1 (0.9%)	1 (0.1%)	0.190
Hypertension	21 (18%)	112 (14%)	0.256	8 (7.4%)	32 (3.3%)	0.052
Diabetes	16 (14%)	47 (5.9%)	0.005	7 (6.5%)	9 (0.9%)	<0.001
Dyslipidemia	15 (13%)	51 (6.4%)	0.018	12 (11%)	34 (3.5%)	0.001
Gout	6 (5.2%)	32 (4.0%)	0.462	3 (2.8%)	0 (0%)	<0.001
Sleep apnea syndrome	4 (3.5%)	7 (0.9%)	0.039	1 (0.9%)	2 (0.2%)	0.271
Fracture	3 (2.6%)	6 (0.7%)	0.092	6 (5.6%)	4 (0.4%)	<0.001

Categorical variables as number (percentage). *p*-values were calculated with Fisher’s exact test for categorical variables.

**Table 3:** Psychiatric comorbidities of participants with schizophrenia and participants without schizophrenia according to sex.

Characteristic	Men			Women		
	Schizophrenic Participants, N = 115	Non-Schizophrenic Participants, N = 801	*p*-Value	Schizophrenic Participants, N = 108	Non-Schizophrenic Participants, N = 975	*p*-Value
Depressive symptoms (CES-D ≥ 8)			<0.001			<0.001
Absent	42 (37%)	618 (77%)		25 (23%)	691 (71%)	
Present	73 (63%)	183 (23%)		83 (77%)	284 (29%)	
Sleep time			<0.001			<0.001
<5 h	10 (8.7%)	87 (11%)		15 (14%)	113 (12%)	
6–7 h	59 (51%)	594 (74%)		50 (46%)	704 (72%)	
8–9 h	33 (29%)	111 (14%)		33 (31%)	150 (15%)	
≥10 h	13 (11%)	9 (1.1%)		10 (9.3%)	8 (0.8%)	
Hypnagogic disorder			<0.001			<0.001
<3 times per week	75 (65%)	688 (86%)		66 (61%)	787 (81%)	
≥3 times per week	40 (35%)	113 (14%)		42 (39%)	188 (19%)	
Deep sleep disorder			<0.001			0.005
<3 times per week	52 (45%)	532 (66%)		60 (56%)	675 (69%)	
≥3 times per week	63 (55%)	269 (34%)		48 (44%)	300 (31%)	
Middle wakening, or early wakening			<0.001			<0.001
<3 times per week	78 (68%)	703 (88%)		62 (57%)	802 (82%)	
≥3 times per week	37 (32%)	98 (12%)		46 (43%)	173 (18%)	
Perceived stress (PSS-4)	9.6 (3.2)	7.1 (2.8)	<0.001	9.9 (3.0)	7.3 (2.8)	<0.001
*Ikigai*			<0.001			<0.001
Present	49 (43%)	500 (62%)		48 (44%)	628 (64%)	
Absent	66 (57%)	301 (38%)		60 (56%)	347 (36%)	
Happiness			<0.001			<0.001
Present	42 (37%)	494 (62%)		56 (52%)	697 (71%)	
Absent	73 (63%)	307 (38%)		52 (48%)	278 (29%)	
Internet use time per week (h)	26 (24)	21 (21)	0.035	20 (22)	17 (19)	0.147

Continuous variables were expressed as mean (SD), and categorical variables as number (percentage). *p*-values were calculated with the Welch Two-Sample *t*-test for continuous variables and with Fisher’s exact test for categorical variables. Abbreviations: CES-D, a modified 11-item Center for Epidemiological Studies Depression Scale; PSS-4, a 4-item Perceived Stress Scale.

**Table 4:** Social comorbidities of participants with schizophrenia and participants without schizophrenia according to sex.

Characteristic	Men			Women		
	Schizophrenic Participants, N = 115	Non-Schizophrenic Participants, N = 801	*p*-Value	Schizophrenic Participants, N = 108	Non-Schizophrenic Participants, N = 975	*p*-Value
Taking regular medical checkups	52 (45%)	536 (67%)	<0.001	43 (40%)	527 (54%)	0.006
Educational background			<0.001			0.003
Junior/senior high school	51 (44%)	220 (27%)		43 (40%)	251 (26%)	
University, junior college, vocational school	64 (56%)	581 (73%)		65 (60%)	724 (74%)	
Occupation			<0.001			<0.001
Unemployed	58 (50%)	119 (15%)		36 (33%)	66 (6.8%)	
Homemaker	3 (2.6%)	5 (0.6%)		29 (27%)	257 (26%)	
White-collar workers	21 (18%)	453 (57%)		25 (23%)	482 (49%)	
Blue-collar workers	33 (29%)	224 (28%)		18 (17%)	170 (17%)	
Type of employment			<0.001			<0.001
Regular	14 (26%)	489 (72%)		5 (12%)	343 (53%)	
Non-regular	30 (56%)	103 (15%)		34 (79%)	274 (42%)	
Self-employed/business people	10 (19%)	85 (13%)		4 (9.3%)	35 (5.4%)	
Household income (million Japanese yen)			<0.001			<0.001
<3	61 (53%)	148 (18%)		47 (44%)	211 (22%)	
3–6	40 (35%)	280 (35%)		40 (37%)	375 (38%)	
6–9	12 (10%)	197 (25%)		16 (15%)	231 (24%)	
≥9	2 (1.7%)	176 (22%)		5 (4.6%)	158 (16%)	
Marital status			<0.001			0.004
Unmarried	88 (77%)	286 (36%)		48 (44%)	371 (38%)	
Married	21 (18%)	463 (58%)		41 (38%)	519 (53%)	
Divorced	6 (5.2%)	37 (4.6%)		14 (13%)	67 (6.9%)	
Widowed	0 (0%)	2 (0.2%)		2 (1.9%)	10 (1.0%)	
Others	0 (0%)	13 (1.6%)		3 (2.8%)	8 (0.8%)	
Family structure						
Living alone	22 (19%)	177 (22%)	0.546	11 (10%)	191 (20%)	0.018
Living with parents	73 (63%)	194 (24%)	<0.001	50 (46%)	229 (23%)	<0.001
Living with spouse	21 (18%)	456 (57%)	<0.001	41 (38%)	512 (53%)	0.004
Living with children	15 (13%)	257 (32%)	<0.001	32 (30%)	348 (36%)	0.243
Living with other people	27 (23%)	50 (6.2%)	<0.001	17 (16%)	91 (9.3%)	0.042
Social support (ESSI)	21 (7)	22 (8)	0.262	22 (7)	23 (7)	0.007
Social capital			0.046			0.014
Less cognitive social capital	70 (61%)	404 (50%)		58 (54%)	400 (41%)	
Less structural social capital	99 (86%)	678 (85%)		90 (83%)	858 (88%)	

Continuous variables were expressed as mean (SD), and categorical variables as number (percentage). *p*-values were calculated with the Welch Two-Sample *t*-test for continuous variables and with Fisher’s exact test for categorical variables. Abbreviations: ESSI, the ENRICHD Social Support Instrument.

## Data Availability

Raw data supporting reported results of this study are human research participant data and are not publicly available but could be made available upon justified requests and after appropriate procedures, including approval from the institutional ethics review committee.

## References

[1] He Y., Tanaka A., Kishi T., Li Y., Matsunaga M., Tanihara S., Iwata N., Ota A. (2022). Recent findings on subjective well-being and physical, psychiatric, and social comorbidities in individuals with schizophrenia: A literature review. Neuropsychopharmacol. Rep..

[2] The Ministry of Health, Labour and Welfare (2017). The Patient Survey in Japan, 2017. https://www.mhlw.go.jp/toukei/saikin/hw/kanja/17/index.html.

[3] The Ministry of Health, Labour and Welfare (2004). The Vision for Reforming Mental Health Care and Welfare. https://www.mhlw.go.jp/topics/2004/09/dl/tp0902-1a.pdf.

[4] Maki S., Nagai K., Ando S., Tamakoshi K. (2021). Structure and predictors of in-hospital nursing care leading to reduction in early readmission among patients with schizophrenia in Japan: A cross-sectional study. PLoS ONE.

[5] Šprah L., Dernovšek M.Z., Wahlbeck K., Haaramo P. (2017). Psychiatric readmissions and their association with physical comorbidity: A systematic literature review. BMC Psychiatry.

[6] Kessler T., Lev-Ran S. (2019). The association between comorbid psychiatric diagnoses and hospitalization-related factors among individuals with schizophrenia. Compr. Psychiatry.

[7] Suzuki Y., Yasumura S., Fukao A., Otani K. (2003). Associated factors of rehospitalization among schizophrenic patients. Psychiatry Clin. Neurosci..

[8] Mitchell A.J., Vancampfort D., Sweers K., van Winkel R., Yu W., De Hert M. (2013). Prevalence of Metabolic Syndrome and Metabolic Abnormalities in Schizophrenia and Related Disorders—A Systematic Review and Meta-Analysis. Schizophr. Bull..

[9] Mamakou V., Thanopoulou A., Gonidakis F., Tentolouris N., Kontaxakis V. (2018). Schizophrenia and type 2 diabetes mellitus. Psychiatriki.

[10] Etchecopar-Etchart D., Korchia T., Loundou A., Llorca P.-M., Auquier P., Lançon C., Boyer L., Fond G. (2021). Comorbid Major Depressive Disorder in Schizophrenia: A Systematic Review and Meta-Analysis. Schizophr. Bull..

[11] Wulff K., Dijk D.-J., Middleton B., Foster R.G., Joyce E.M. (2012). Sleep and circadian rhythm disruption in schizophrenia. Br. J. Psychiatry.

[12] Crespo-Facorro B., Such P., Nylander A.-G., Madera J., Resemann H.K., Worthington E., O’Connor M., Drane E., Steeves S., Newton R. (2021). The burden of disease in early schizophrenia—A systematic literature review. Curr. Med. Res. Opin..

[13] Green M.F., Kern R.S., Braff D.L., Mintz J. (2000). Neurocognitive deficits and functional outcome in schizophrenia: Are we measuring the “right stuff”?. Schizophr. Bull..

[14] Sugai T., Suzuki Y., Yamazaki M., Shimoda K., Mori T., Ozeki Y., Matsuda H., Sugawara N., Yasui-Furukori N., Minami Y. (2016). High Prevalence of Obesity, Hypertension, Hyperlipidemia, and Diabetes Mellitus in Japanese Outpatients with Schizophrenia: A Nationwide Survey. PLoS ONE.

[15] Baba K., Guo W., Chen Y., Nosaka T., Kato T. (2022). Burden of schizophrenia among Japanese patients: A cross-sectional national health and wellness survey. BMC Psychiatry.

[16] Leucht S., Burkard T., Henderson J., Maj M., Sartorius N. (2007). Physical illness and schizophrenia: A review of the literature. Acta Psychiatr. Scand..

[17] Buckley P.F., Miller B.J., Lehrer D.S., Castle D.J. (2009). Psychiatric comorbidities and schizophrenia. Schizophr. Bull..

[18] Mueser K.T., McGurk S.R. (2004). Schizophrenia. Lancet.

[19] Schoepf D., Uppal H., Potluri R., Heun R. (2014). Physical comorbidity and its relevance on mortality in schizophrenia: A naturalistic 12-year follow-up in General Hospital admissions. Eur. Arch. Psychiatry Clin. Neurosci..

[20] Abdullah H.M., Shahul H.A., Hwang M.Y., Ferrando S. (2020). Comorbidity in Schizophrenia: Conceptual Issues and Clinical Management. Focus Am. Psychiatr. Publ..

[21] Evensen S., Wisløff T., Lystad J.U., Bull H., Ueland T., Falkum E. (2016). Prevalence, Employment Rate, and Cost of Schizophrenia in a High-Income Welfare Society: A Population-Based Study Using Comprehensive Health and Welfare Registers. Schizophr.Bull..

[22] Lin D., Kim H., Wada K., Aboumrad M., Powell E., Zwain G., Benson C., Near A.M. (2022). Unemployment, homelessness, and other societal outcomes in patients with schizophrenia: A real-world retrospective cohort study of the United States Veterans Health Administration database: Societal burden of schizophrenia among US veterans. BMC Psychiatry.

[23] Bouwmans C., de Sonneville C., Mulder C.L., Hakkaart-van Roijen L. (2015). Employment and the associated impact on quality of life in people diagnosed with Schizophrenia. Neuropsychiatr. Dis. Treat..

[24] Tabuchi T., Shinozaki T., Kunugita N., Nakamura M., Tsuji I. (2019). Study Profile: The Japan “Society and New Tobacco” Internet Survey (JASTIS): A Longitudinal Internet Cohort Study of Heat-Not-Burn Tobacco Products, Electronic Cigarettes, and Conventional Tobacco Products in Japan. J. Epidemiol..

[25] Kusama T., Kiuchi S., Takeuchi K., Ikeda T., Nakazawa N., Kinugawa A., Osaka K., Tabuchi T. (2022). Information Usage and Compliance with Preventive Behaviors for COVID-19: A Longitudinal Study with Data from the JACSIS 2020/JASTIS 2021. Healthcare.

[26] Wakabayashi M., Sugiyama Y., Takada M., Kinjo A., Iso H., Tabuchi T. (2022). Loneliness and Increased Hazardous Alcohol Use: Data from a Nationwide Internet Survey with 1-Year Follow-Up. Int. J. Environ. Res. Public Health.

[27] Sasaki R., Ota A., Yatsuya H., Tabuchi T. (2022). Gender Difference in Fear and Anxiety about and Perceived Susceptibility to COVID-19 in the Third Wave of Pandemic among the Japanese General Population: A Nationwide Web-Based Cross-Sectional Survey. Int. J. Environ. Res. Public Health.

[28] The Ministry of Health, Labour and Welfare (1991). The Standards for the Degree of Independent Living for Elderly and Disabled People. https://www.mhlw.go.jp/file/06-Seisakujouhou-12300000-Roukenkyoku/0000077382.pdf.

[29] Foebel A.D., Pedersen N.L. (2016). Genetic Influences on Functional Capacities in Aging. Gerontologist.

[30] DeSalvo K.B., Bloser N., Reynolds K., He J., Muntner P. (2006). Mortality prediction with a single general self-rated health question. J. Gen. Intern. Med..

[31] Radloff L.S. (1977). The CES-D Scale: A Self-Report Depression Scale for Research in the General Population. Appl. Psychol. Meas..

[32] Kohout F.J., Berkman L.F., Evans D.A., Cornoni-Huntley J. (1993). Two shorter forms of the CES-D (Center for Epidemiological Studies Depression) depression symptoms index. J. Aging Health.

[33] Warttig S.L., Forshaw M.J., South J., White A.K. (2013). New, normative, English-sample data for the short form perceived stress scale (PSS-4). J. Health Psychol..

[34] García H., Miralles F. (2017). Ikigai: The Japanese Secret to a Long and Happy Life.

[35] The ENRICHD Investigators (2001). Enhancing Recovery in Coronary Heart Disease (ENRICHD) study intervention: Rationale and design. Psychosom. Med..

[36] The ENRICHD Investigators (2000). Enhancing recovery in coronary heart disease patients (ENRICHD): Study design and methods. Am. Heart J..

[37] Grootaert C., Narayan D., Jones V.N., Woolcock M. (2004). Measuring Social Capital: An Integrated Questionnaire.

[38] Murayama H., Fujiwara Y., Kawachi I. (2012). Social capital and health: A review of prospective multilevel studies. J. Epidemiol..

[39] The Ministry of Internal Affairs and Communications (2021). White Paper 2021 on Information and Communications in Japan. https://www.soumu.go.jp/johotsusintokei/whitepaper/ja/r03/html/nd242120.html.

[40] Hjorthøj C., Stürup A.E., McGrath J.J., Nordentoft M. (2017). Years of potential life lost and life expectancy in schizophrenia: A systematic review and meta-analysis. Lancet Psychiatry.

[41] Brown S., Kim M., Mitchell C., Inskip H. (2010). Twenty-five year mortality of a community cohort with schizophrenia. Br. J. Psychiatry.

[42] Virtanen T., Eskelinen S., Sailas E., Suvisaari J. (2017). Dyspepsia and constipation in patients with schizophrenia spectrum disorders. Nord. J. Psychiatry.

[43] Koizumi T., Uchida H., Suzuki T., Sakurai H., Tsunoda K., Nishimoto M., Ishigaki T., Goto A., Mimura M. (2013). Oversight of constipation in inpatients with schizophrenia: A cross-sectional study. Gen. Hosp. Psychiatry.

[44] Xu Y., Amdanee N., Zhang X. (2021). Antipsychotic-Induced Constipation: A Review of the Pathogenesis, Clinical Diagnosis, and Treatment. CNS Drugs.

[45] Deng C. (2013). Effects of antipsychotic medications on appetite, weight, and insulin resistance. Endocrinol. Metab. Clin. N. Am..

[46] Sankaranarayanan A., Johnson K., Mammen S.J., Wilding H.E., Vasani D., Murali V., Mitchison D., Castle D.J., Hay P. (2021). Disordered Eating among People with Schizophrenia Spectrum Disorders: A Systematic Review. Nutrients.

[47] Otsuka R., Tamakoshi K., Yatsuya H., Murata C., Sekiya A., Wada K., Zhang H.M., Matsushita K., Sugiura K., Takefuji S. (2006). Eating fast leads to obesity: Findings based on self-administered questionnaires among middle-aged Japanese men and women. J. Epidemiol..

[48] Ohi K., Shimada T., Kuwata A., Kataoka Y., Okubo H., Kimura K., Yasuyama T., Uehara T., Kawasaki Y. (2019). Smoking Rates and Number of Cigarettes Smoked per Day in Schizophrenia: A Large Cohort Meta-Analysis in a Japanese Population. Int. J. Neuropsychopharmacol..

[49] The Ministry of Health, Labour and Welfare (2019). The National Health and Nutrition Survey in Japan, 2019. https://www.mhlw.go.jp/stf/seisakunitsuite/bunya/kenkou_iryou/kenkou/eiyou/r1-houkoku_00002.html.

[50] Stubbs B., Gaughran F., Mitchell A.J., De Hert M., Farmer R., Soundy A., Rosenbaum S., Vancampfort D. (2015). Schizophrenia and the risk of fractures: A systematic review and comparative meta-analysis. Gen. Hosp. Psychiatry.

[51] Stubbs B., Mueller C., Gaughran F., Lally J., Vancampfort D., Lamb S.E., Koyanagi A., Sharma S., Stewart R., Perera G. (2018). Predictors of falls and fractures leading to hospitalization in people with schizophrenia spectrum disorder: A large representative cohort study. Schizophr. Res..

[52] Kishimoto T., De Hert M., Carlson H.E., Manu P., Correll C.U. (2012). Osteoporosis and fracture risk in people with schizophrenia. Curr. Opin. Psychiatry.

[53] Stubbs B., Vancampfort D., Veronese N., Solmi M., Gaughran F., Manu P., Rosenbaum S., De Hert M., Fornaro M. (2016). The prevalence and predictors of obstructive sleep apnea in major depressive disorder, bipolar disorder and schizophrenia: A systematic review and meta-analysis. J. Affect. Disord..

[54] Takahashi K.I., Shimizu T., Sugita T., Saito Y., Takahashi Y., Hishikawa Y. (1998). Prevalence of sleep-related respiratory disorders in 101 schizophrenic inpatients. Psychiatry Clin. Neurosci..

[55] Leung R.S., Bradley T.D. (2001). Sleep apnea and cardiovascular disease. Am. J. Respir. Crit. Care Med..

[56] Rossom R.C., Hooker S.A., O’Connor P.J., Crain A.L., Sperl-Hillen J.M. (2022). Cardiovascular Risk for Patients with and without Schizophrenia, Schizoaffective Disorder, or Bipolar Disorder. J. Am. Heart Assoc..

[57] Polcwiartek C., Loewenstein D., Friedman D.J., Johansson K.G., Graff C., Sørensen P.L., Nielsen R.E., Kragholm K., Torp-Pedersen C., Søgaard P. (2021). Clinical Heart Failure Among Patients with and without Severe Mental Illness and the Association with Long-Term Outcomes. Circ. Heart Fail..

[58] He Q., You Y., Yu L., Yao L., Lu H., Zhou X., Wu S., Chen L., Chen Y., Zhao X. (2020). Uric acid levels in subjects with schizophrenia: A systematic review and meta-analysis. Psychiatry Res..

[59] Zhuo C., Triplett P.T. (2018). Association of Schizophrenia with the Risk of Breast Cancer Incidence: A Meta-analysis. JAMA Psychiatry.

[60] Chou F.H.-C., Tsai K.-Y., Wu H.-C., Shen S.-P. (2016). Cancer in patients with schizophrenia: What is the next step?. Psychiatry Clin. Neurosci..

[61] Conley R.R., Ascher-Svanum H., Zhu B., Faries D.E., Kinon B.J. (2007). The Burden of Depressive Symptoms in the Long-Term Treatment of Patients with Schizophrenia. Schizophr. Res..

[62] Hawton K., Sutton L., Haw C., Sinclair J., Deeks J.J. (2005). Schizophrenia and suicide: Systematic review of risk factors. Br. J. Psychiatry.

[63] Kaskie R.E., Graziano B., Ferrarelli F. (2017). Schizophrenia and sleep disorders: Links, risks, and management challenges. Nat. Sci. Sleep.

[64] Hacimusalar Y., Karaaslan O., Misir E., Amuk O.C., Hacimusalar G. (2022). Sleep quality impairments in schizophrenia and bipolar affective disorder patients continue during periods of remission: A case-controlled study. Sleep Sci..

[65] Hou C.-L., Li Y., Cai M.-Y., Ma X.-R., Zang Y., Jia F.-J., Lin Y.-Q., Ungvari G.S., Chiu H.F.K., Ng C.H. (2017). Prevalence of Insomnia and Clinical and Quality of Life Correlates in Chinese Patients with Schizophrenia Treated in Primary Care. Perspect. Psychiatr. Care.

[66] Nugent K.L., Chiappelli J., Rowland L.M., Hong L.E. (2015). Cumulative Stress Pathophysiology in Schizophrenia as Indexed by Allostatic Load. Psychoneuroendocrinology.

[67] Gutiérrez-Rojas L., González-Domenech P.J., Junquera G., Halverson T.F., Lahera G. (2021). Functioning and Happiness in People with Schizophrenia: Analyzing the Role of Cognitive Impairment. Int. J. Environ. Res. Public Health.

[68] Holubova M., Prasko J., Hruby R., Kamaradova D., Ociskova M., Latalova K., Grambal A. (2015). Coping strategies and quality of life in schizophrenia: Cross-sectional study. Neuropsychiatr. Dis. Treat..

[69] Fervaha G., Agid O., Takeuchi H., Foussias G., Remington G. (2016). Life satisfaction and happiness among young adults with schizophrenia. Psychiatry Res..

[70] Sakai I., Mizuno E. (2011). Purpose of life of people with mental illness living in the community. J. Jpn. Acad. Nurs. Sci..

[71] Lee J.-Y., Chung Y.-C., Song J.-H., Lee Y.-H., Kim J.-M., Shin I.-S., Yoon J.-S., Kim S.-W. (2018). Contribution of stress and coping strategies to problematic Internet use in patients with schizophrenia spectrum disorders. Compr. Psychiatry.

[72] Kim S.-W., Park W.-Y., Jhon M., Kim M., Lee J.-Y., Kim S.-Y., Kim J.-M., Shin I.-S., Yoon J.-S. (2019). Physical Health Literacy and Health-related Behaviors in Patients with Psychosis. Clin. Psychopharmacol. Neurosci..

[73] Burns J.K., Tomita A., Kapadia A.S. (2014). Income inequality and schizophrenia: Increased schizophrenia incidence in countries with high levels of income inequality. Int. J. Soc. Psychiatry.

[74] Tanihara S., Tsuji M., Kawazoe M., Yamanokuchi T., Shimura H. (2017). Comparison of the number of claims per population in each large classification of disease. J. Health Welf. Stat..

[75] The National Federation of Associations of Families with The Mental Illness in Japan National Survey Report on the Promotion of Independent Community Living for Persons with Mental Disabilities and Effective Family Support and Other Measures to Ensure Peace of Mind for Families in 2017. https://fields.canpan.info/report/detail/21126.

[76] Hendryx M., Green C.A., Perrin N.A. (2009). Social support, activities, and recovery from serious mental illness: STARS Study Findings. J. Behav. Health Serv. Res..

[77] De Silva M.J., McKenzie K., Harpham T., Huttly S.R.A. (2005). Social capital and mental illness: A systematic review. J. Epidemiol. Community Health.

[78] APA (2013). Diagnostic and Statistical Manual of Mental Disorders, Fifth Edition (DSM-5).

[79] Waters F., Woodward T., Allen P., Aleman A., Sommer I. (2012). Self-recognition deficits in schizophrenia patients with auditory hallucinations: A Meta-analysis of the literature. Schizophr. Bull..

